# Dichorionic triplets following frozen-thawed poor-stage embryo transfer: a report of two cases and a review

**DOI:** 10.1186/s12958-017-0302-1

**Published:** 2017-10-03

**Authors:** Atsushi Yanaihara, Shirei Ohgi, Kenichirou Motomura, Ryoma Taniguchi, Shota Hatakeyama, Takumi Yanaihara

**Affiliations:** Yanaihara Women’s Clinic, 1-26-29 Ofuna, Kamakura, Kanagawa 247-0056 Japan

**Keywords:** Dichorionic triplet, IVF, Cleavage stage embryo transfer, Blastocyst transfer

## Abstract

**Background:**

We describe two cases of dichorionic triplet pregnancy after a frozen-thawed poor-stage embryo transfer.

**Main body of the abstract:**

A 39-year-old and a 41-year-old woman underwent ART treatment. The first patient underwent intracytoplasmic sperm injection (ICSI) at 34 years of age, and two frozen-thawed poor-stage embryos were transferred at 39 years of age with assisted hatching, resulting in a trichorionic triamniotic triplet pregnancy. The second patient underwent ICSI, and two poor-grade blastocysts were transferred followed by assisted hatching, resulting in a dichorionic triamniotic triplet pregnancy.

In the first case, the heartbeat of one monozygotic twin fetus had stopped on day 48 post-transfer (9 weeks 2 days), resulting in a dichorionic diamniotic twin pregnancy. A healthy boy and girl were delivered by elective caesarean section at 36 weeks, 5-days gestation. In the second case, the patient underwent selective reduction of the monochorionic twins, resulting in a single pregnancy that was vaginally delivered without any problems at 38 weeks 0-days gestation.

**Short conclusions:**

Numerous factors may be associated with the development of a monochorionic pregnancy; however, controversies still remain. The present morphological grading for embryos is insufficient for inhibiting the development of a monochorionic pregnancy.

## Background

In Japan, one in 21 newborns result from in vitro fertilization (IVF) treatment, and the demand for IVF is increasing in various societal backgrounds. Multiple pregnancies are associated with risks during the perinatal period and infertility treatments, such as clomiphene citrate, increase the likelihood of a multiple pregnancy (MP) [1]. Various approaches in infertility treatment have been developed to reduce the incidence of MP. Recent improvements in culturing technology, from the cleavage stage to the blastocyst stage, allow a single embryo transfer, decreasing the risk of an MP [2]. Thus, MPs may become less of a concern. However, even with a single blastocyst transfer, the likelihood of a monozygotic twin pregnancy is reported to be increased compared to that for a natural pregnancy [3, 4]. A dichorionic diamniotic twin pregnancy is suggested to occur when a single embryo splits within 3 days after fertilization. In contrast, a monochorionic diamniotic twin pregnancy occurs when a single embryo splits 4 ~ 7 days after fertilization [5, 6]. The monozygotic twin pregnancy rate has been reported to be 0.4% in Japan (among all pregnancies), while the rate of monozygotic triplet pregnancy is 0.004%. To our knowledge, the dichorionic triamniotic triplet pregnancy rate has not been adequately reported; however, based on simple calculations, the rate appears to be 0.004% (the zygotic twin rate times the monozygotic twin rate); the trichorionic triamniotic triplet pregnancy rate is thought to be similar.

Clinically, pregnancies become difficult with the aging of patients. With older patients, it is often impossible to obtain one good quality embryo; a transfer of more than two embryos is usual. In addition, cases are more likely to involve poor-grade embryo transfers. Herein, we describe two cases of dichorionic triplet pregnancy after frozen-thawed poor-stage embryo transfers. These cases highlight the concerns of transferring multiple embryos of poor grade.

Institutional Review Board approval was obtained for this study.

## Case description

### Case report 1

A 34-year-old woman with 4 years of primary infertility (resulting from male-factor infertility) underwent IVF treatment at our clinic after several failed intrauterine inseminations. A mild stimulation protocol was performed using clomiphene and human menopausal gonadotropin (HMG; 150 units every other day; HMG150; Ferring Pharmaceuticals, Tokyo, Japan) [7]. Oocyte maturation was triggered using 1000 IU of human chorionic gonadotropin (hCG) (HCG 10000 U for injection; Fujipharma, Toyama-shi, Toyama). Nine mature oocytes were collected, of which 7 were fertilized via intracytoplasmic sperm injection (ICSI) and cultured for 3 days. Embryo transfer was performed during the next cycle due to the endometrial thickness. Three day-3 embryos were cryopreserved. During the next cycle, a frozen-thawed embryo transfer was performed and she became pregnant. She delivered a healthy baby at 40 weeks of gestation.

**Fig. 1:**
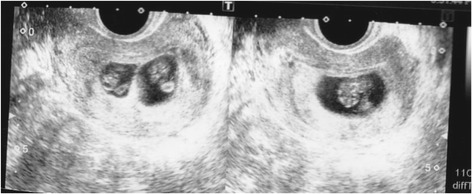
Ultrasound image of a dizygotic trichorionic triamniotic triplet in the seventh week of pregnancy

Wanting another child, she requested a frozen embryo transfer when she was 39 years old. The endometrium was prepared with hormone replacement therapy (HRT). The two remaining embryos were of poor grade (Veeck’s classification: 9 cells grade 4 and 8 cells grade 4, respectively) and were transferred using assisted hatching under transabdominal ultrasound (US). Luteal progesterone support was administered for 2 weeks. The woman successfully conceived, as confirmed by an hCG level of 450 IU/L on day 11 post-transfer (4 weeks 0 days). HRT was continued and a transvaginal US was performed on day 18 post-transfer (5 weeks 0 days). There were two gestational sacs (GSs) with dizygotic twins suspected. On day 32 post-transfer (7 weeks 0 days), an US showed two GSs with three fetuses, and a dizygotic trichorionic triamniotic triplet pregnancy was suspected (Fig. 1). On day 48 post-transfer (9 weeks 2 days), the heartbeat of one of the monozygotic twin fetuses had stopped, resulting in a dichorionic diamniotic twin pregnancy. The subsequent pregnancy course went well, and a healthy boy and girl were delivered by elective caesarean section at 36 weeks 5 days of gestation.

### Case report 2

**Fig. 2:**
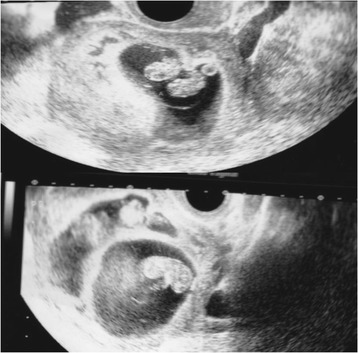
Ultrasound image of a dichorionic triamniotic triplet in the seventh week of pregnancy

The patient was a 41-year-old woman who complained of secondary infertility for 3 years related to male-factor infertility. She had a regular menstrual cycle and all routine analyses for infertility were unremarkable. She underwent IVF treatment. A mild stimulation protocol was followed for 9 days, with clomiphene and HMG (150 units every other day; HMG150; Ferring Pharmaceuticals, Tokyo, Japan) [7]. Thirty-five hours before egg collection, we administered 1000 IU of hCG (HCG 10000 U for injection; Fujipharma, Toyama-shi, Toyama). Six mature oocytes were collected, all of which were fertilized using ICSI, yielding 4 viable embryos on day 3 (Veeck’s classification: 10 cells grade 2, 9 cells grade 2, 8 cells grade 2, and 9 cells grade 3, respectively). Transfer was performed during the next cycle due to the endometrium thickness. A single day-3 embryo (Veeck’s classification: 10 cells grade 2) was transferred during an HRT cycle, but the transfer was not successful. In the following cycle, the culture was extended from a day-3 embryo to a day-5 blastocyst. The endometrium was prepared with HRT and two embryos (among the three remaining cleavage stage embryos) were grown to blastocysts. Assisted hatching was performed, and 2 blastocysts (Gardner’s classification: 5CB and 3CC, respectively) were transferred under transabdominal US guidance. Luteal progesterone support was given for 2 weeks. The serum hCG level was 520 IU/L on day 9 post-transfer (4 weeks 0 days). Transvaginal US performed at 5 weeks, 0-days gestation showed 2 GSs inside the uterus. At 7 weeks 0-days gestation, 3 fetal heart beats were detected and a dichorionic triamniotic triplet pregnancy (dichorionic monoamniotic twin + single) was suspected (Fig. 2). After being informed of the maternal and fetal risks, the patient decided to proceed with selective reduction, hoping to obtain a single pregnancy. At 9 weeks 2-days gestation, reduction was successfully performed. The subsequent pregnancy course went well and the remaining single fetus was vaginally delivered without any problems at 38 weeks 0 days.

## Discussion

MPs are well-known to be associated with high risks; the membranous diagnosis of an MP is very important for determining the obstetrical prognosis. Several factors, such as patient history [8], maternal age [9], ovarian stimulation [10], and in vitro culture condition [11], have been reported to influence the incidence of MPs in advanced reproductive technologies. Furthermore, numerous reports have implicated procedures, such as the micromanipulation of the zona pellucida, assisted embryo hatching, and ICSI [8, 12–15], as factors related to MP. However, other studies suggest these factors may not be independent causes of MP [16–20]. Although extending the culture from the cleavage stage to the blastocyst stage may increase the incidence of MP [11, 19], a recent study suggested that blastocyst transfers are not associated with an increased rate of monozygotic twin pregnancy when controlling for embryo cohort quality [21]. Given the complexity of an MP event, several factors may be associated with the underlying mechanisms [22].

Maternal age and hereditary components are the best-defined determinants for spontaneous multiple births [23]. Thus, the germinal weakness of the egg may be the issue, rather than the age of the material itself. In addition, cell junction or cell adhesion in the inner cell mass (ICM) is inferred to decrease with age, becoming fragile [24, 25]. However, the stability of the cell fusion may also be poor in MPs, which has nothing to do with age. In the blastocyst stage, a poor ICM grade is associated with a significantly higher incident of monochorionic diamniotic twinning [26]. Moreover, becoming pregnant via IVF treatment, rather than spontaneously, indicates poor stability of the embryo itself from the beginning. Further studies are needed to resolve the mechanisms involved in the increased risk of MP in IVF treatment.

In both of the present cases, the transferred embryos were of morphologically poor grade, similar to those in the report by Tal et al. [20]. Two embryos were transferred, as the likelihood of pregnancy was considered to be extremely low for a single embryo transfer due to the embryo quality. There are no previous cases concerning MP with a poor-grade cleavage stage single embryo transfer. Thus, it is unclear whether the multiple embryo transfer alone caused the MP. Would this phenomenon occur in a poor-grade cleavage single embryo transfer? Although embryo quality is known to play the most important role in pregnancy, the opposite appeared true in the present cases. This may be due to the environment of the IVF system. For example, Anifandis et al. [27] reported that the prolonged culture condition affects gene expression in pre-implantation embryos derived from an in vitro culture system. During the various ART procedures, the gamete epigenomes are exposed to external stress factors that influence the establishment and maintenance of genomic imprinting.

In a previous study, a regression analysis revealed that both the blastocyst grade and the distribution of mosaic abnormal cells were significantly correlated with the likelihood of being diagnosed with an MP via an array comparative genome hybridization performed on clinical trophectoderm biopsies [28]. Moreover, the authors have provided suggestions for improved laboratory and clinical management of blastocyst stage Preimplantation Genetic Screening (PGS) cycles; as commonly used parameters of blastocyst evaluation are inadequate to allow improved selection among euploid embryos [29]. However, reports evaluating the relationship between cleavage stage embryo quality and molecular karyotyping are lacking, as a risk of diagnostic error exists.

The onset of time-lapse technology, which allows one to observe a time series of the macroscopic findings of the embryo quality, has made a big impact in the field of obstetrics [30]. For example, an MP may be predicted by time-lapse technology, via the observation of the inner cell mass separating. Moreover, a relationship between embryo metabolism and viability has been established. The result of the study by Minasi et al. [31] regarding ongoing pregnancy and miscarriage rates suggests that embryo evaluation by PGS or time-lapse imaging may not improve IVF outcomes. In contrast, as a routine diagnosis in IVF clinics, molecular karyotyping via comparative genomic hybridization arrays or next generation sequencing, together with morphokinetic data, is now being considered for the creation of more robust algorithms for embryo selection [32, 33], potentially reducing the incidence of MPs [26].

## Conclusions

In conclusion, the current report emphasizes the difficulty in avoiding an MP given a poor selection of candidate embryos. An egg that is immature or is hard to grow naturally can result in a pregnancy with IVF/ICSI treatment. Unfortunately, the current techniques do not sufficiently allow the selection of a strong egg or an egg that does not miscarry. Before transferring the embryo in IVF treatment, morphological embryo selection with three-dimensional time-lapse technology may reduce the incidence of MP.

## Abbreviations

GS(s): Gestational sac(s)
hCG: Human chorionic gonadotropin
HMG: Human menopausal gonadotropin
HRT: Hormone replacement therapy
ICM: Inner cell mass
ICSI: Intracytoplasmic sperm injection
IVF: In vitro fertilization
MP(s): Multiple pregnancy(s)
PGS: Preimplantation genetic screening
US: Ultrasound
